# Supplementary material for: Thiophenium Salts as New Oxidant for Redox Polymerization under Mild- and Low-Toxicity Conditions
Source: Molecules. 2023 Jan 7;28(2):627. doi: 10.3390/molecules28020627 (PMC9861688; doi:10.3390/molecules28020627)

Supporting information

Thiophenium salts as new Oydant for RedOx polymerization under mild conditions

Alexis BARRAT[1,3], Frédéric SIMON[3], Jérôme MAZAJCZYK[3], Bruno CHARRIERE[4], Stéphane FOUQUAY[3], Jacques LALEVEE[1,2]

1: Université de Haute-Alsace, CNRS, IS2M UMR 7361, F-68100 Mulhouse, France.

2: Université de Strasbourg, F-67081, France.

3: Bostik Smart Technology Centre, F-60280 Venette, France.

4: Arkema, F92700 Colombes, France.

I. Synthesis of 2,8-ditertiobutyl-5 (trifluoromethyl)dibenzothiophenium trifluoromethanesulfonate (Thiophenium (I))

In a 25 mL two necks round bottom flask is added 0.5 g of sodium triflinate (1 eq., 3 mmol) under anhydrous and argon atmosphere conditions followed by 10 mL of anhydrous nitromethane as solvent. After total dissolution, is added 1 ml of triflic anhydride (2 eq., 6 mmol). After stirring for 10 min, the 4,4'-Di-tert-butylbiphenyl (1 eq., 3 mmol, 0.799 g) is added as a solution by dissolution in 5 mL of nitromethane. The reaction is monitored by TLC using cyclohexane as eluent.

After 24h, the nitromethane is distilled off under reduced pressure and several washing were performed with toluene (4*3 mL) to remove nitromethane as much as possible.

The crude is then diluted is 3,5 mL of distilled water followed by 3,5 mL of diethyl ether causing the precipitation of the product after one night of stirring.

After filtration and several washing with glacial diethyl ether (4*3 mL), the product is dried under reduced pressure at 40°C giving a white solid (0.167 g, 21%).

Thiophenium (I) H-NMR in $CDCl_3$

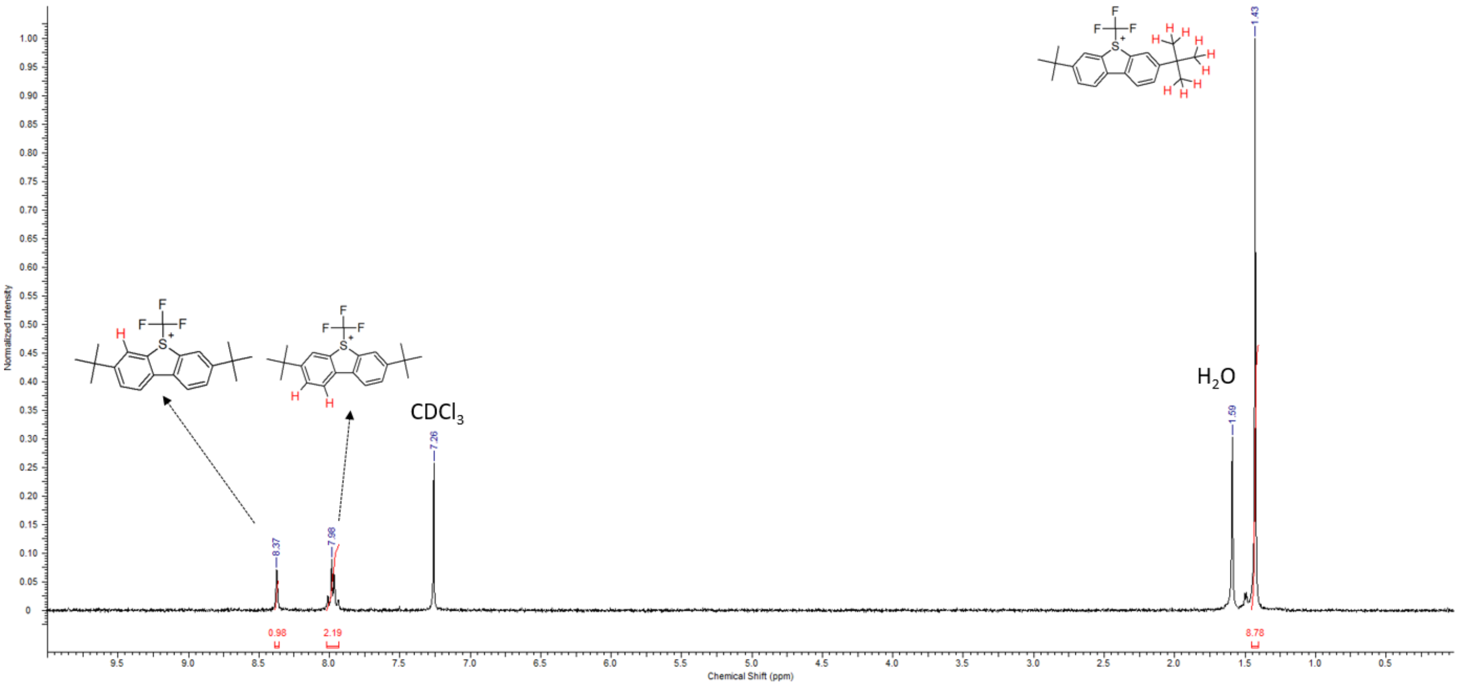

Thiophenium (I) F-NMR in $CDCl_3$

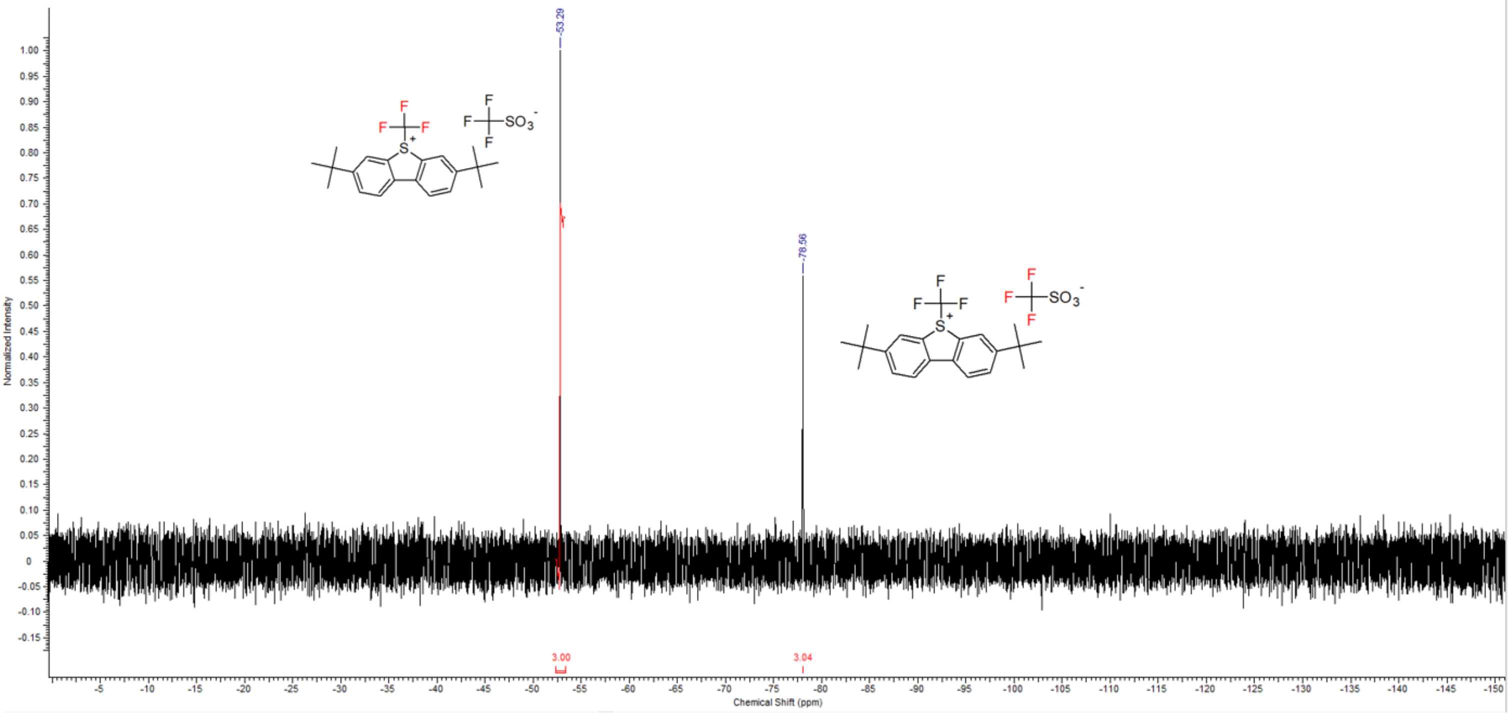

II. Synthesis of 2,8-difluoro-S-(trifluoromethyl)dibenzothiophenium hexafluorophosphate (Thiophenium IV)

In a 10 mL two necks round bottom flask was added 0.192 g of sodium hexafluorophosphate (1 eq., 1,14 mmol) followed by 5 mL of acetonitrile as the solvent. After complete dissolution, 0.5 g of 2,8-Difluoro-5 (Trifluoromethyl)dibenzothiophenium trifluoromethanesulfonate (Thiophenium (II)) (1 eq., 1,14 mmol) was added. The mixture was heated at 60°C for 2 hours.

After evaporation of acetonitrile under reduced pressure, the crude was dissolved in 10 mL of dichloromethane and washed with a saturated solution of $NaHCO_3$(3*5 mL). The dichloromethane was distilled off, 5 mL of acetonitrile was added and a large excess of diethyl ether was added until precipitation of the product occurred (approx. 20 mL). The product was obtained as a white powder (0.413 g, 83%).

Thiophenium (IV) H-NMR in DMSO

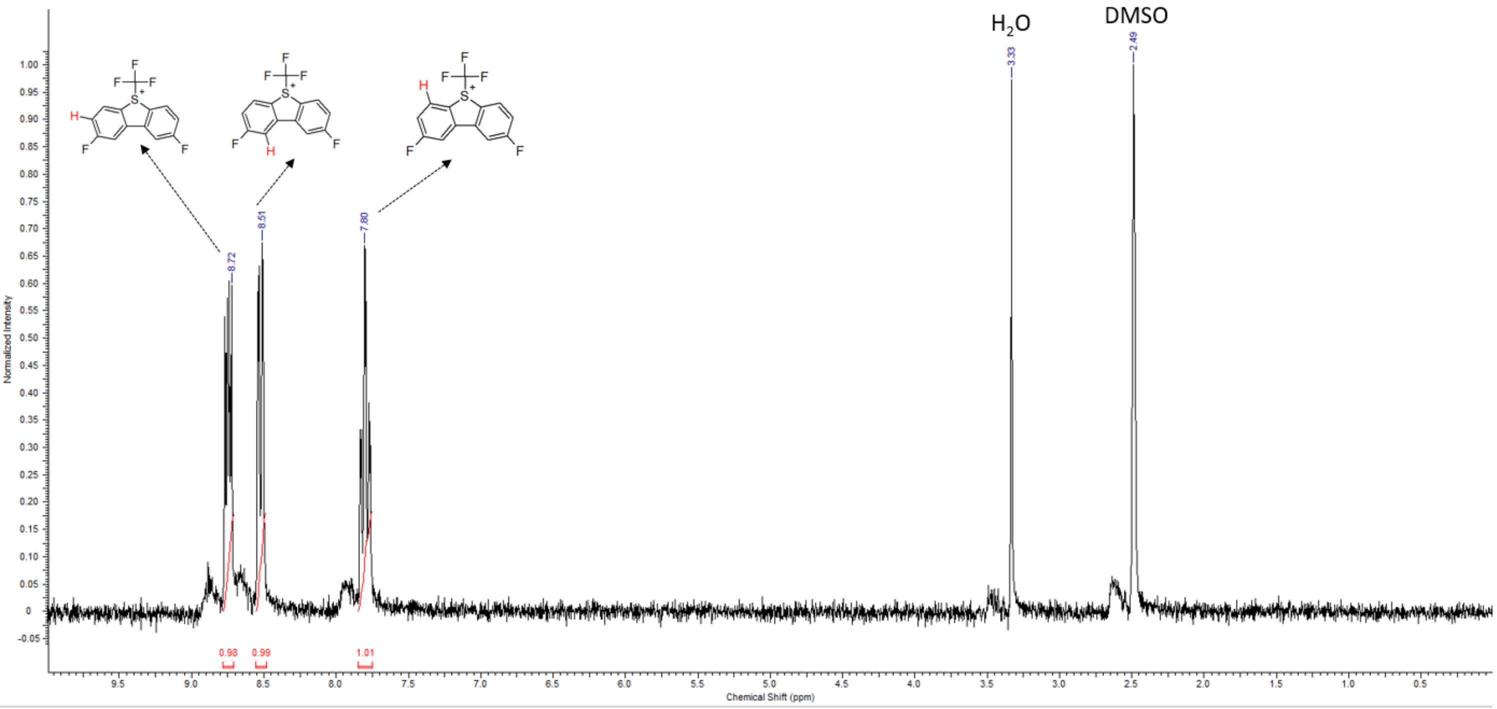

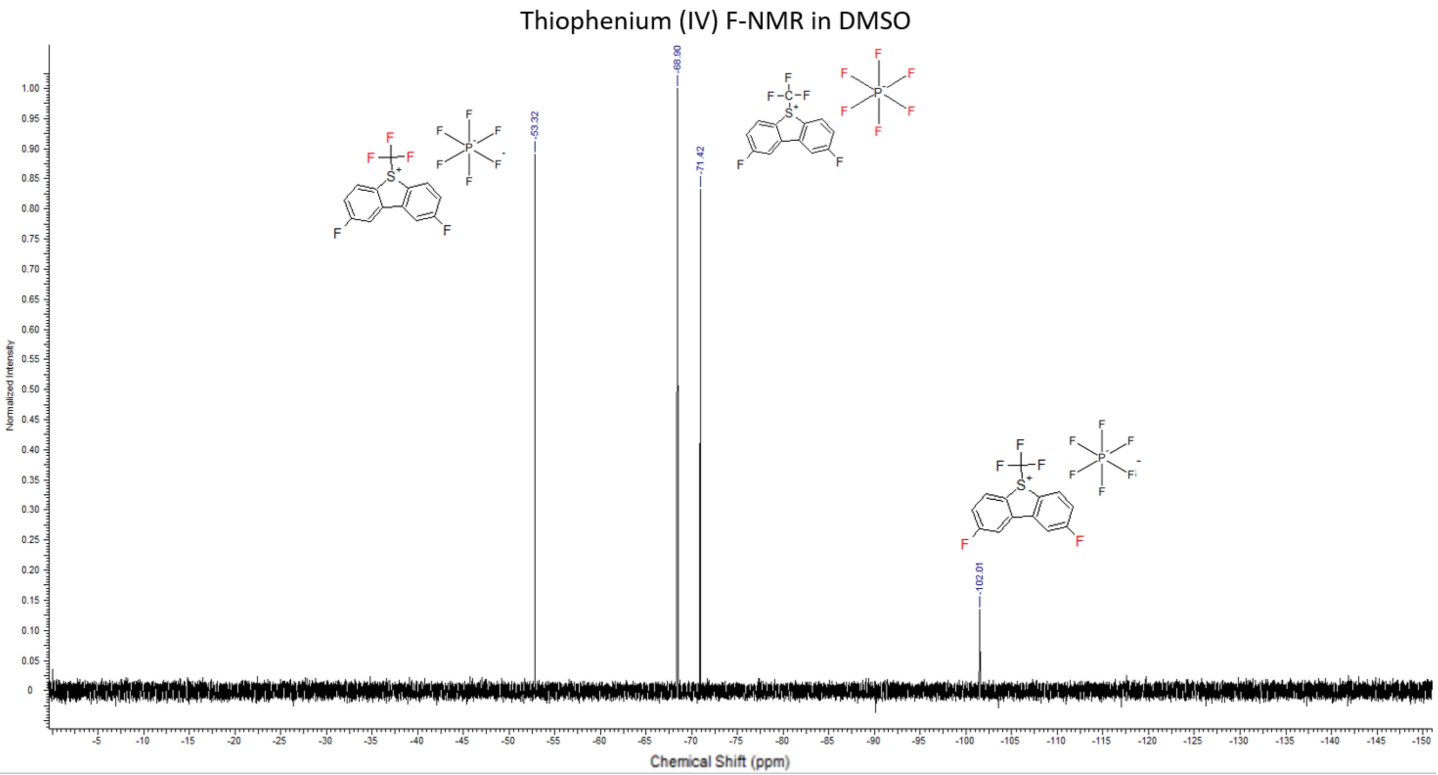

III. UV-Visible spectrum of Thiophenium (III) salt

using a Jasco V-730 spectrophotometer with a Thiophenium (III) solution at 10-3 mol.L-1 I acetonitrile. Measures were conducterd in a Quartz cuvette of 1 cm².

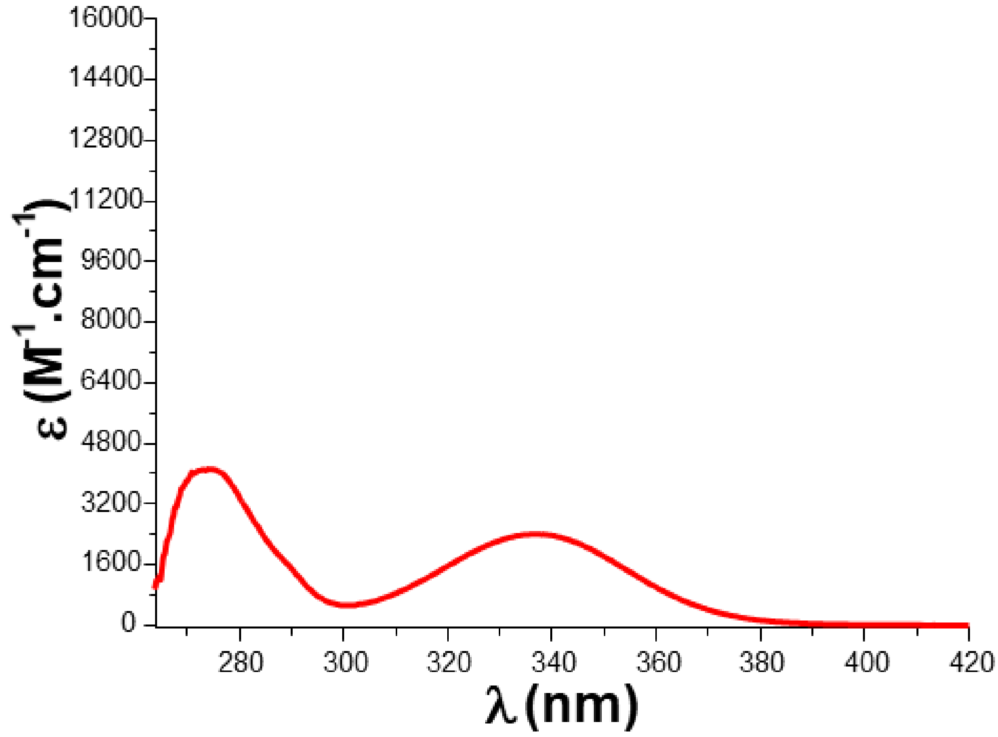

Supplement: Supplementary file 1 [file molecules-28-00627-s001.zip › molecules-2074877-supplementary.pdf]
